# Impact of athletic profiles and the relative age effect on the future achievement levels of young basketball players

**DOI:** 10.3389/fspor.2025.1616800

**Published:** 2025-09-12

**Authors:** Yannis Irid, Jean-Claude Pineau, Quentin De Larochelambert, Jean-François Toussaint, Adrien Sedeaud

**Affiliations:** ^1^ IRMES—URP 7329, Institut de Recherche Médicale et D'Épidémiologie du Sport, Université de Paris, Paris, France; ^2^ Institut National du Sport, de L'Expertise et de la Performance (INSEP), Paris, France; ^3^ Fédération Française de Basketball, Paris, France; ^4^ Centre National de la Recherche Scientifique (CNRS), Paris, France

**Keywords:** athletic profiling, relative age effect, youth basketball, talent identification, long-term athletic development

## Abstract

Talent identification in youth sports is a multifactorial process, with athletic profiling and the Relative Age Effect (RAE) playing critical roles. However, few studies have investigated their combined influence on long-term success in youth basketball. This study explores how athletic profiles and RAE influence future success in young male basketball players. A total of 131 players (age: 14.5 ± 0.7 years; height: 180.6 ± 9.7 cm) were assessed on speed, agility, vertical jump, and endurance. RAE was evaluated via birth quarter distribution. A principal component analysis followed by hierarchical clustering revealed four distinct performance profiles: Hybrid (*n* = 45), Elevated (*n* = 34), Resilient (*n* = 35), and Explosive (*n* = 19). These clusters were cross-referenced with players’ future levels of competition: Amateur (*n* = 105), Developmental (*n* = 19), and Elite (*n* = 9). Both athletic profiles and RAE showed statistically noteworthy associations with future achievement (*p* < 0.10). Notably, players in the Resilient cluster—characterized by lower physical test scores and later birthdates—were more likely to reach the Elite level. These findings highlight the importance of accounting for individual developmental trajectories and relative age in talent identification. They support a more holistic, long-term approach to player evaluation, challenging the assumption that early physical superiority reliably predicts future elite status.

## Introduction

Basketball is an immensely popular sport, played by millions worldwide across diverse age groups and levels of expertise. A player's success depends on a range of physical and physiological attributes, including height, aerobic and anaerobic capacities, strength, agility, and speed, among others (1, 2). Identifying athletes with the potential to excel at the highest levels requires an in-depth understanding of these key factors that underpin the selection and development of young players.

Height is one of the most significant factors in basketball (3). At elite levels, both height and body mass contribute significantly to enhanced physical and team performance (4, 5). Strategically, shorter players often excel in rapid ball movement and perimeter play, while taller players leverage their size near the basket for effective shooting and rebounding (2, 6). In the National Basketball Association (NBA), a correlation between height and scoring underscores the importance of this attribute (7). Thus, an athlete's anthropometric profile is a critical determinant of higher-level performance in basketball (7, 8). Along with anthropometric data, other performance-related variables such as jumping ability are central to talent evaluation, as they facilitate better shooting opportunities, rebounding, and shot-blocking (9–12).

Basketball also places significant demands on both aerobic and anaerobic energy systems (13). Rule changes and game pacing have increased cardiovascular requirements, with noted differences among positions—for example, point guards often show the highest maximal heart rates compared to shooting guards or centers (14–17). These differences underscore the importance of anaerobic capacity for high-intensity actions and adequate aerobic fitness for faster recovery between bouts, training sessions, and games (18–21). Consequently, tests measuring jumping, sprinting, aerobic capacity, and neuromuscular performance are commonly used in talent identification programs (22–26).

A recent review highlights the complex interplay between anthropometry, physiology, and physical performance in early basketball talent identification; notably, the Relative Age Effect (RAE) was not routinely integrated into these assessments (27). The RAE refers to a biased distribution of birth dates within the same age group, where athletes born earlier in the selection year often have higher success and advancement rates (28). In basketball, various studies document its presence. For instance, among French basketball players aged 7–18 years, birthdate distribution influenced selection and development (29). Another study suggested a link between RAE and higher dropout rates, aligning with findings in other sports such as swimming (30, 31).

Together, athletic profiling and the RAE shape early developmental pathways and influence access to competitive opportunities. Yet, little is known about how their combined impact predicts long-term achievement. This study therefore investigates whether specific physical performance profiles and birth quarter distribution are associated with future success among young basketball players. Understanding these influences can help refine talent-identification methods and optimize development strategies in youth basketball.

## Materials and methods

### Participants

A total of 131 male basketball players (age: 14.5 ± 0.7 years; height: 180.6 ± 9.7 cm) participated in comprehensive physical testing under the supervision of the French Basketball Federation's technical staff.

### Physical tests

The testing battery included measurements of stature, maximal aerobic speed (MAS), sprint performance, basketball-specific agility (Butterfly test), and vertical jump height:

• **Stature**: measured in centimeters in a standard standing position.
• **Maximal Aerobic Speed** (MAS): determined using the Luc Léger 20 m shuttle run test, a progressive and intermittent running protocol with audio cues.
• **Sprint**: time (in seconds) recorded over a 10-meter distance from a standing start, using a stopwatch.
• **Butterfly Test**: a pre-planned agility circuit performed around the basketball paint area, with directional changes marked by cones at each corner; the best time (in seconds) from two trials was recorded.
• **Vertical Jump**: participants stood approximately 30 cm from a wall and marked their maximal reach with chalk. Three maximal jumps were performed, and the best result was used to compute jump height as the difference between standing and jumping reach (in cm).

### Performance levels

Future performance levels were retrospectively stratified into three categories based on players’ achievements in adulthood:

• **Category 1 (Elite)**: participation in top-level competition (NBA or European professional leagues).
• **Category 2 (Developmental)**: still enrolled in a recognized development program by the age of 18.
• **Category 3 (Amateur)**: participation in lower-level competitions or dropout from sport.

Stratification into these categories was performed retrospectively after players turned 18, ensuring that the classification reflected post-youth development outcomes.

### Statistical analysis

#### Classification of players

A Principal Component Analysis (PCA) was conducted on five variables: stature, Butterfly test time, 10 m sprint time, MAS, and vertical jump height. An ascending hierarchical clustering on principal components (HCPC) using Ward's method (32) was then applied to the first two PCA axes to group athletes into performance-based clusters.

#### Relative age effect

Players were categorized into four groups based on their birthdates: those born from January 1 to March 31 were assigned to the first quarter (Q1), from April 1 to June 30 to the second quarter (Q2), from July 1 to September 30 to the third quarter (Q3), and from October 1 to December 31 to the fourth quarter (Q4).

To assess the presence of a Relative Age Effect (RAE) in the overall sample, regardless of other variables, a chi-square test of goodness-of-fit to a uniform distribution was performed. This test evaluated whether the distribution of birth quarters deviated from a uniform distribution, under the assumption of an equal number of births in each quarter. Cramér's V was also calculated to quantify effect size and provide insight into the strength of the association between observed and expected distributions. A Cramér's V of 0.10 indicates a small effect, 0.30 a medium effect, and 0.50 a large effect, providing a framework for interpretation of the observed associations (33).

A separate chi-square test of independence was conducted to examine the relationship between birth quarters and performance clusters. To further explore significant associations, standardized (Haberman) residuals (34) were analyzed to identify which cells contributed most to the observed effects.

Given the exploratory nature of this study and the relatively small sample size, a significance threshold of *p* < 0.10 was adopted. This more permissive threshold is commonly used in preliminary or hypothesis-generating research to detect potential trends that may warrant further investigation (35). In addition, *post hoc* power analyses were conducted for the main statistical tests to evaluate the ability of the study to detect effects of the observed magnitude. When observed power was low, and effect size estimates were imprecise (e.g., wide confidence intervals), these limitations were explicitly acknowledged and considered when interpreting the findings.

### Ethics statement

This study was approved by the scientific committee of the Institut de Recherche Médicale et d’Épidémiologie du Sport. Data collection complied with the General Data Protection Regulation (GDPR) and adhered to the principles of the Declaration of Helsinki. All data were anonymized prior to analysis.

## Results

### Player classification

**Figure 1:**
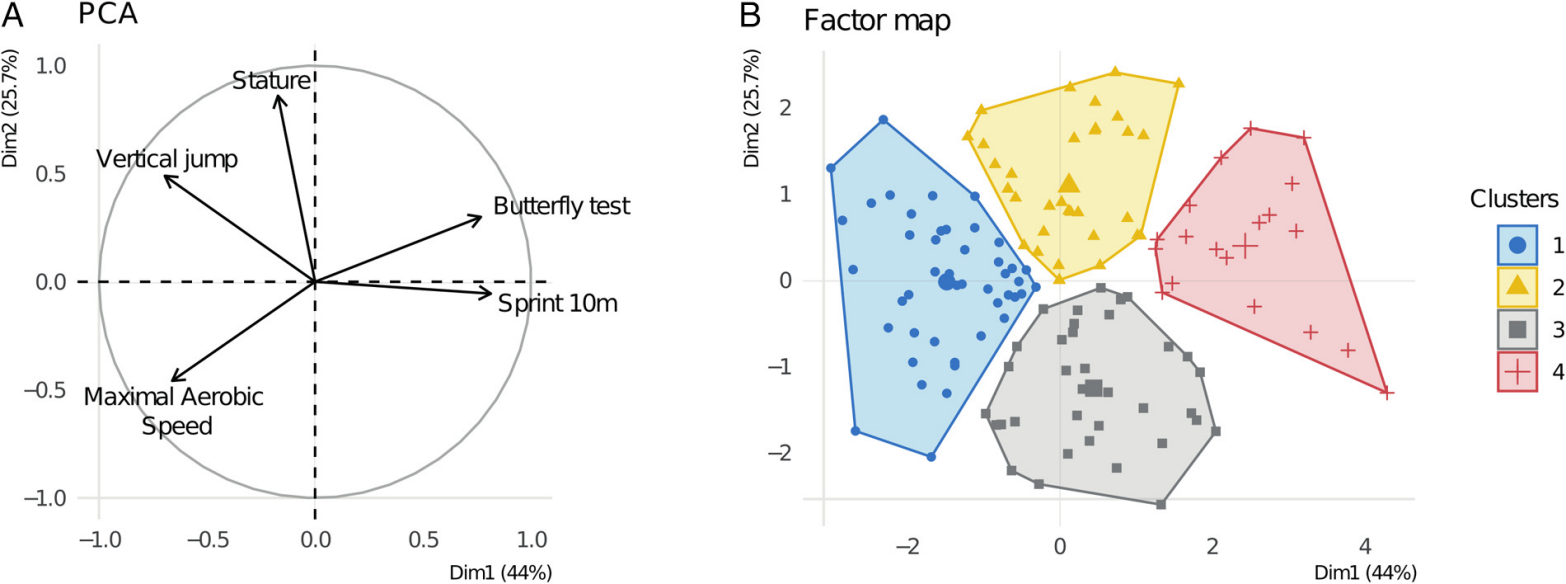
**(A)** PCA—distribution of performance variables on the first factorial plane. **(B)** Cluster Distribution on the First Factorial Plane of the PCA.

Figure 1A presents the first factorial plane of the PCA, which accounts for 69.7% of the total variance. The first principal axis (44.0%) contrasts 10-meter sprint and Butterfly test performance (negative loading) with vertical jump height and MAS (positive loading), while stature does not contribute significantly. The second axis (25.6%) contrasts stature and jump height (positive loading) with MAS (negative loading); 10 m sprint is not strongly represented on this axis.

Figure 1B shows the four clusters obtained through HCPC.

• Cluster 1 (“Hybrid”): high MAS and jump height, but lower sprint and agility (Butterfly) performance.
• Cluster 2 (“Elevated”): taller players with good jumping ability and relatively low MAS.
• Cluster 3 (“Resilient”): shorter players with lower jump performance but good MAS.
• Cluster 4 (“Explosive”): strong sprint and agility performance, but lower jump height and MAS.

### Overall relative Age

A Chi-square goodness-of-fit test comparing the distribution of birth quarters to a uniform distribution revealed a significant relative age effect across the entire sample (*p* = 0.0005, *χ*^2^ = 31.9). The observed effect size was moderate [Cramér's V = 0.35, 95% CI (0.21, 0.46)]. A *post hoc* power analysis indicated a high observed power (87%) to detect the effect, suggesting that the test was sufficiently powered despite the modest sample size (*N* = 87).

Q1 was markedly overrepresented (resid = + 5.51), while Q3 (resid = −2.66) and Q4 (resid = −2.17) were underrepresented, indicating a clear skew in birth date distribution favoring early-born players.

### Performance clusters, future achievement, and relative age effect

#### Clusters and future performance levels

**Figure 2:**
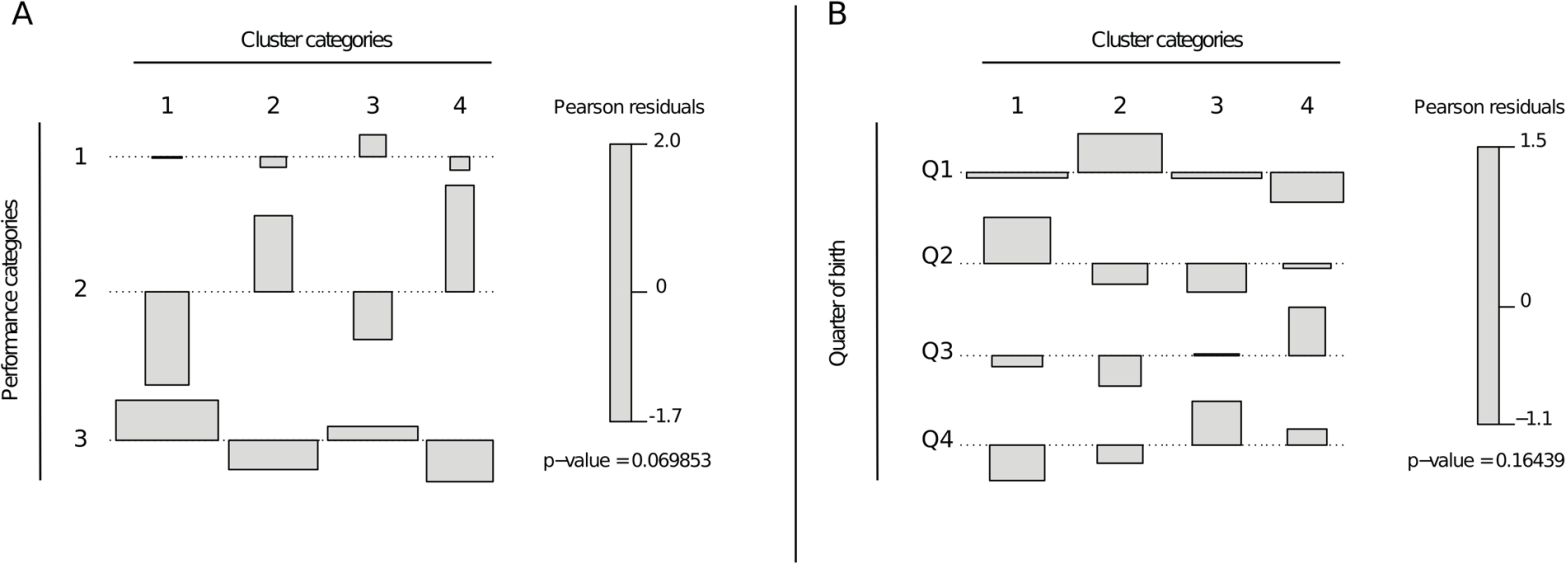
**(A)** Distribution of players by performance category across clusters. **(B)** Distribution of Players by Birth Quarter Across Clusters.

A significant association was observed between player clusters and their future performance levels (*χ*^2^ = 11.6, *p* < 0.10; Cramér's V = 0.21). In Cluster 1 (“Hybrid”), Category 2 players were underrepresented (resid = −2.31), and Category 3 players overrepresented (resid = 2.01). Cluster 4 (“Explosive”) showed the opposite trend, with an overrepresentation of Category 2 players (resid = 2.32) and fewer Category 3 players than expected (resid = −1.82). Cluster 2 (“Elevated”) also included more Category 2 players than expected (resid = 1.76). Cluster 3 (“Resilient”) had the highest proportion of Category 1 players (Figure 2A).

Although this association reached statistical significance at *α* = 0.10, the *post hoc* power analysis revealed a relatively low observed power (52%) to detect the observed effect size [Cramér's V = 0.21, 95% CI (0.00, 0.29)]. This wide confidence interval reflects substantial uncertainty around the strength of the association, likely due to the modest sample size (*N* = 133). The limited statistical power and imprecise effect estimate represent important limitations, and the findings should therefore be interpreted with caution.

#### Clusters and relative age

No statistically significant association was found between cluster distribution and players’ birth quarters [*χ*^2^ = 12.9, *p* = 0.165; Cramér's V = 0.22, 95% CI (0.00, 0.28)]. However, descriptive trends suggested localized deviations from uniformity, with a higher proportion of Q2 players in Cluster 1 and more Q1 players in Cluster 2 (Figure 2B). A *post hoc* power analysis revealed a low observed power (36%), indicating limited sensitivity to detect small to moderate effects given the modest sample size (*N* = 87). Consequently, the absence of a significant global association may reflect insufficient statistical power rather than a true lack of effect, and the observed local patterns should be interpreted with caution.

## Discussion

This study explored how relative age and athletic profiles interact to shape long-term success trajectories in youth basketball. Two main findings emerge (1): early physical advantages are not consistently predictive of elite-level achievement, and (2) despite an overall RAE, relatively younger players (born later in the year) are more frequently found among the elite performers.

### Profiles and RAE

The results confirm the presence of a RAE in youth basketball, consistent with previous research (36–39). Players born in Q1 and Q2—especially within the “Explosive” and “Hybrid” clusters—displayed superior physical performance in sprint, agility, jump height, and aerobic capacity. This advantage likely stems from more advanced maturation levels, reinforcing the well-documented maturation-selection hypothesis (29, 36, 40–43). Such results illustrate how being born earlier in the selection year can yield immediate benefits in competition and talent selection processes.

Interestingly, these physical advantages did not translate into a clear pathway toward elite performance. This phenomenon, sometimes termed the “underdog effect” (44), has been noted in sports like rugby union (45), cricket (46), and football (47). Several plausible mechanisms could drive this effect, including psychological factors, identity construction in the face of adversity, and enhanced learning or resilience among late-born athletes (46, 48). Additionally, it is possible that coaches subconsciously give preferential treatment to those with early physical advantages, thus overlooking potentially talented but physically less mature players (49). These findings collectively underscore the necessity of refining selection criteria to account for not just chronological age but also relative, biological, and training ages (50). In doing so, basketball federations and coaches can ensure a more equitable talent development system that looks beyond immediate physical attributes, ultimately fostering a deeper pool of potential elite athletes.

Moreover, recognizing the interplay between relative age and the dynamic trajectories of player development enriches current talent identification practices. Taking RAE into account helps mitigate biases that might prematurely exclude later-born athletes from advanced training opportunities. Moving forward, this more nuanced perspective opens new avenues for research that investigate how RAE-driven imbalances manifest in different stages of a basketball career—from regional and national development programs to professional and international competitions.

### Profiles and physical characteristics

**Figure 3:**
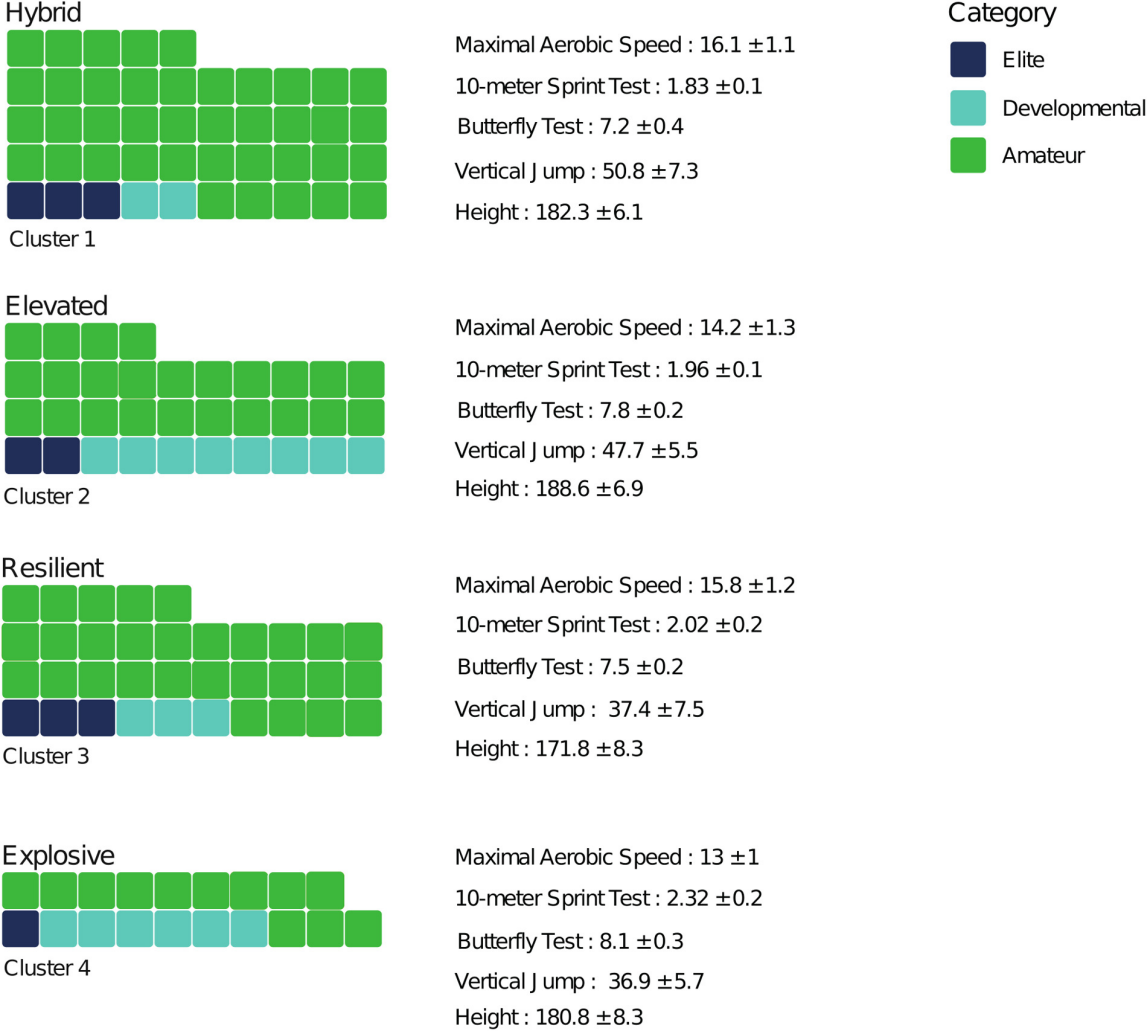
Distribution of players among clusters and their physical performances.

The identification of four distinct athletic profiles—“Explosive,” “Hybrid,” “Elevated,” and “Resilient”—offers further insight into the limitations of relying solely on early physical performance to predict long-term success (Figure 3).

For instance, although the “Explosive” cluster showed strong speed and agility at a young age, its members predominantly remained at the amateur or developmental level by age 18. These observations align with a recent review on basketball talent identification (27) indicating that while top youth performers display superior anthropometric, physiological, and physical characteristics (51), such early advantages do not necessarily predict senior success (52). Indeed, across multiple Olympic sports, junior performance explains only a minor fraction (2.2%) of the reliable variance in senior performance (52). Thus, progression rates and annual improvements—rather than absolute performance at a young age—may serve as more reliable talent predictors (50, 53, 54).

Interestingly, the “Hybrid” profile—characterized by higher MAS and jump height—showed stronger representation within the amateur category. This observation contrasts with existing literature, which typically associates higher aerobic capacity (VO_2_ max) with enhanced recovery and sustained high-intensity efforts, favoring progression in basketball (27). Similarly, superior aerobic fitness has been reported in the most promising young basketball players (55). This discrepancy could be explained by several factors such as insufficient technical-tactical development despite favorable physical attributes. Consequently, while endurance and repeated-effort capacity remain important, they should be interpreted within a broader developmental context, rather than as standalone predictors of success.

Meanwhile, the “Elevated” cluster—characterized by taller stature and robust vertical jump performance—showed a relatively stronger pathway to the developmental category. Height remains a well-known asset in basketball (42, 56, 57), often forming the basis of early selection (27, 58). Research on professional leagues, such as the NBA, corroborates that height and body composition hold pivotal roles in offensive and defensive skills, including shooting, passing, rebounding, and shot-blocking (1, 2, 6). However, our results also emphasize that height alone is insufficient: the combination of height and lower-limb power (e.g., strong vertical jump) appears key in identifying players with greater developmental potential. This finding aligns with various studies demonstrating that higher jumping ability and lower-body power—often measured via the Squat Jump (SJ) or Countermovement Jump (CMJ)—are essential for basketball success (27, 59).

Collectively, these observations reflect the multifaceted nature of talent identification in basketball. While certain physical attributes (e.g., speed, agility) may confer early advantages and selection benefits, they do not reliably guarantee sustained success (52). This highlights the need for holistic evaluation frameworks—encompassing not only physical performance but also psychological, technical, and tactical factors (50). Coaches and talent scouts should adopt broader, more flexible selection criteria, accounting for individual developmental pathways and future growth potential, rather than focusing solely on immediate measures of performance. Importantly, variables such as mental resilience, learning ability, and willingness to adapt should be integrated into monitoring and evaluation programs, acknowledging that players mature and evolve at different rates.

Moreover, placing an emphasis on relative, biological, and training ages can help capture each player's developmental status more accurately (50). Given that physical and psychological attributes can shift dramatically between early adolescence and adulthood, continuous longitudinal tracking is vital for making informed decisions regarding training regimens, positional roles, and competitive opportunities. By refining these frameworks, youth basketball programs can balance current performance assessments with the recognition that some late maturers—often overlooked in conventional selection models—may ultimately become top-tier players.

### Limitations

Key limitation of this study lies in the relatively small number of players who reached the elite category (*n* = 9), which limits the statistical power and generalizability of the findings. While effect sizes were included to aid interpretation, larger and more diverse samples are needed to confirm the observed patterns and enhance external validity.

Additionally, the exclusive focus on physical parameters provides only a partial view of basketball potential. Other key domains—such as technical skills, tactical understanding, and psychological traits—were not assessed, yet play a critical role in long-term success.

Further longitudinal research is warranted to track how early advantages or disadvantages evolve across adolescence and into adulthood. Moreover, future work should consider recalibrating performance metrics by incorporating relative age, biological maturity, and training experience, all of which could improve the precision and fairness of youth talent assessments.

## Conclusion

This study sheds light on the interplay between athletic profiles and relative age in shaping long-term outcomes in youth basketball. While certain physical attributes confer early advantages, they do not reliably predict adult success. Notably, an “underdog effect” emerged, with relatively younger players—despite lower physical scores at age 14—more often reaching the elite level.

These findings challenge conventional talent identification practices that prioritize early physical dominance. A more holistic approach, integrating developmental trajectories, resilience, and non-physical attributes, may better support equitable and effective talent development.

## Data Availability

The raw data supporting the conclusions of this article will be made available by the authors, without undue reservation.
